# Surgical Skills Course for Fourth Year Medical Students Entering an Orthopaedic Surgery Residency

**DOI:** 10.15694/mep.2020.000034.1

**Published:** 2020-02-19

**Authors:** Lucy E. Meyer, Ocean V. Thakar, Symone M. Brown, Jeffrey D. Trojan, Mary K. Mulcahey

**Affiliations:** 1 Tulane University School of Medicine; 2 Drexel University College of Medicine

**Keywords:** surgical skills, orthopaedic surgical skills, medical students, orthopaedic surgery

## Abstract

This article was migrated. The article was marked as recommended.

Surgical skills training outside of the operating room is a critical component of surgical education. There has been little incorporation of these programs for medical students entering orthopaedic surgery residencies. As such, there is concern that incoming orthopaedic residents matriculate with skills below residency programs’ expectations. This study aimed to assess the need for an orthopaedic surgical skills course during the 4 ^th^ year of medical school.An anonymous electronic survey was emailed to 1457orthopaedic surgery residents and 732 current orthopaedic residency applicants using application data from a single orthopaedic residency program during the 2015-2016 through 2017-2018 cycles. 200 resident and 31 applicant emails were returned undeliverable, resulting in distribution to 1257 residents and 701 applicants. 135 junior residents (11% response rate) and 181 applicants (26% response rate) completed portions of the survey. 76 of 117 (65%) residents and 170 of 181 (94%) applicants did not participate in a formal orthopaedic surgical skills course. 110 of 118 (93%) residents and 160 of 171 (94%) applicants indicated that implementing such a course before entering residency would be beneficial. Applicants rated basic fracture reduction (95%; 171/180), casting/splinting (94%; 170/180), and reading basic x-rays (90%; 162/180) as the most important potential course components.Most respondents were not exposed to an orthopaedic skills course prior to residency. Participants indicated that such a course would be beneficial to incoming orthopaedic residents. Pilot programs should be developed to optimize an orthopaedic preparatory skills course for 4 ^th^ year medical students pursuing careers in orthopaedic surgery.

## Introduction

Surgical skills training outside of the operating room is a critical component of surgical education and has been widely implemented in surgical residency training curricula ( Zeng, Woodhouse and Brunt, 2010). However, there has been little to no incorporation of these programs for senior medical students entering surgical residencies ( Zeng, Woodhouse and Brunt, 2010).The majority of medical students’ exposure to surgery stems from 3 ^rd^ year general surgery clerkships with varying experiences within and between different institutions (Amini *et al.,* 2016). Although some basic surgical skills training occurs in the operating room, medical students have few opportunities to develop their technical skills during clinical clerkship years (Amini *et al.,* 2016). Given the lack of standardization in undergraduate surgical education, there is growing concern that 4 ^th^ year medical students matriculating into surgical residencies graduate with skills that are below the expectations of residency programs (Amini *et al.,* 2016).

In 2004, the American Surgical Association (ASA) Blue Ribbon Committee evaluated medical student surgical education within the United States and found that there was a need for students to increase their preparedness for surgical residency during the final year of medical school ( Tocco *et al.,* 2013). The ASA reported that the biggest hindrance to student preparation was an absence of structure within the 4 ^th^ year curriculum ( Debas *et al.,* 2005). The ASA believes not utilizing this time for surgical skills training represents a lost opportunity in medical student education. They concluded that a formal 4 ^th^ year surgical skills course, along with increased availability of faculty mentorship, would sustain medical student interest in general surgery and improve preparation for surgical residency ( Debas *et al.,* 2005).

As a result, the American College of Surgeons (ACS) Division of Education sought to standardize surgical education for students entering general surgery residency. In doing so, the ACS designated a core set of fundamental skills that senior medical students should obtain prior to residency and using this framework, multiple institutions created preparatory courses for students ( *Successfully Navigating the First Year of Surgical Residency,* 2005). Studies evaluating the effectiveness of such programs have reported significant improvements in student skill and confidence while performing intern-level responsibilities, including managing acute on-call problems, emergent and non-emergent bedside procedures, basic suturing, and knot tying ( Zeng, Woodhouse and Brunt, 2010), ( Karam *et al.,* 2013), ( Karam *et al.,* 2013), ( Westerlind *et al.,* 2014). In 2009, Zeng *et al*. evaluated the efficacy of Washington University College of Medicine’s general surgery preparatory program for 4 ^th^ year medical students ( Zeng, Woodhouse and Brunt, 2010). Using data from the first four years of the program (2006-2009), the authors found that the average time spent on each task, which included knot tying, central line placement, intubation, chest tube placement, and basic laparoscopy, as well as the total time for all the tasks were significantly decreased at the end of the course when compared to pre-course results (p<0.001). Moreover, students reported increased post-course confidence in completing these tasks compared to pre-course ( Zeng, Woodhouse and Brunt, 2010). Recognizing the effectiveness of these individual programs, the ACS released a universal curriculum in 2015 currently being pilot tested in 47 institutions nationwide. The ACS aims to eventually create a standardized program to be implemented at all U.S. medical schools that will appropriately train senior students in the basic skills necessary for surgical residency ( Naylor *et al.,* 2010).

Similarly, a study by Karam *et al*. surveyed orthopaedic residency program directors (PDs) and residents regarding a potential surgical skills curriculum for orthopaedic surgery interns. Survey responses demonstrated an overwhelming consensus for the need to incorporate a PGY-1 surgical skills curriculum focused on basic skills and the use of simulation technology ( Peyre *et al.,* 2006). In July 2013, the Accreditation Council for Graduate Medical Education (ACGME) and the American Board of Orthopaedic Surgery (ABOS) mandated that residency programs implement a formal, curriculum-based training program for PGY-1 residents ( Teo *et al.,* 2011).Numerous studies have demonstrated the efficacy of these surgical skills training programs ( American Board of Surgery *et al.,* 2014), ( Dougherty and Marcus, 2013). Butler *et al*. implemented a didactic and simulation-based educational module to train PGY-1s and MS4s on closed reduction and percutaneous pinning of pediatric supracondylar humeral fractures ( Butler *et al.,* 2017). After the course, an evaluation of knowledge surrounding management of supracondylar fractures showed no significant difference between the interns and sub-interns who completed the module and a group of PGY-2 to PGY-5 orthopaedic residents.Furthermore, interns and PGY-2 to PGY-5 residents did not differ in a post-course skills test fractures ( Butler *et al.,* 2017).

Given the success of 4 ^th^ year general surgery training programs and the orthopaedic module reported by Butler *et al*., senior medical students entering orthopaedic residency may benefit from a 4 ^th^ year orthopaedic skills preparatory course fractures ( Butler *et al.,* 2017). The purpose of this study was to survey 4 ^th^ year medical students pursuing orthopaedic surgery and junior orthopaedic residents to determine the necessity and usefulness of a 4 ^th^ year orthopaedic surgical skills training course.

## Methods

After receiving Internal Review Board (IRB) approval from the senior author’s institution, 2 surveys were created via SurveyMonkey (San Mateo, CA). One survey was designed specifically for medical students applying to orthopaedic surgery, and the other was designed for junior orthopaedic surgery residents (PGY1 and PGY2). Junior orthopaedic surgery residents were particularly chosen due to potential similarities in surgical skills competency with 4 ^th^ year medical students. Moreover, junior residents who have recently graduated medical school are better equipped to assess the training needs of 4 ^th^ year students when compared to their senior resident peers. Surveys consisted of questions regarding the participants’ medical school demographics, exposure to an orthopaedic surgical skills course during medical school, and opinions about the utility and implementation of such a course. Surveys were distributed via email to all individuals who applied to the orthopaedic surgery residency program at our institution from the 2015-2016 academic year to the 2017-2018 academic year (Supplementary File 1). Email addresses were obtained by reviewing archived residency applications for 2189 applicants. Surveys were sent to recipients beginning in February 2018, with reminder emails sent at 2 weeks and 4 weeks after initial request. Descriptive statistics were utilized to analyze the applicant and resident survey responses.

## Results/Analysis

### Demographic Characteristics

#### Orthopaedic Surgery Residency Applicants

Thirty-one applicant emails were returned as undeliverable, resulting in a total of 701 surveys distributed to current 4 ^th^ year medical students. Of the 701 applicants who received the survey, 181 responded (response rate of 26%). 171 of 181 applicants (95%) were 4 ^th^ year medical students. The 10 applicant respondents who were not 4 ^th^ year students were either already residents (2), a post-graduate (1), a research fellow (1), a re-applicant (1), graduates (4), and skipped this question (1). All demographic results seen in Table 1.

#### Current Junior Orthopaedic Surgery Residents

Two-hundred resident emails were returned as undeliverable, resulting in distribution of 1257 surveys to orthopaedic surgery residents. Of the 1257 PGY1 and PGY2 residents who presumably received the survey, 138 responded (response rate 11%). Further demographic details demonstrated in Table 1.

**Table 1.** Respondent Demographics.

	Applicants	Number (%)	Residents	Number (%)
**Age (years)**	21-24	2 (1.1)	21-25	0 (0.0)
25-28	152 (84.0)	26-30	96 (82.1)	
29-32	18 (9.9)	31-35	19 (16.2)	
33+	9 (5.0)	36+	2 (1.7)	
**Sex**	Male	147 (81.2)	Male	90 (76.9)
Female	34 (18.8)	Female	27 (23.1)	
**Geographic Location of Medical School (%)**	Northeast - New England (CT, ME, MA, NH, RI, VT)	10 (5.6)	Northeast - New England (CT, ME, MA, NH, RI, VT)	6 (5.1)
Northeast - Middle Atlantic (NJ, NY, PA)	26 (14.5)	Northeast - Middle Atlantic (NJ, NY, PA)	23 (19.7)	
Midwest - East North Central (IN, IL, MI, OH, WI)	20 (11.2)	Midwest - East North Central (IN, IL, MI, OH, WI)	22 (18.8)	
Midwest - West North Central (IA, KS, MN, MO, NE, ND, SD)	15 (8.4)	Midwest - West North Central (IA, KS, MN, MO, NE, ND, SD)	8 (6.8)	
South - South Atlantic (DE, DC, FL, GA, NC, SC, VA, WV)	45 (25.1)	South - South Atlantic (DE, DC, FL, GA, NC, SC, VA, WV)	19 (3.4)	
South - East South Central (AL, KY, MS, TN)	22 (12.3)	South - East South Central (AL, KY, MS, TN)	4 (3.4)	
South - West South Central (AR, LA, OK, TX)	31 (17.3)	South - West South Central (AR, LA, OK, TX)	20 (17.1)	
West - Mountain (AZ, CO, ID, NM, MT, UT, NV, WY)	5 (2.8)	West - Mountain (AZ, CO, ID, NM, MT, UT, NV, WY)	8 (6.8)	
West - Pacific (AK, CA, HI, OR, WA)	5 (2.8)	West - Pacific (AK, CA, HI, OR, WA)	7 (6.0)	
**Year in School/Program**	MS1	0 (0.0)	PGY-1	59 (50.0)
MS2	0 (0.0)	PGY-2	47 (48.3)	
MS3	0 (0.0)	PGY-3	2 (1.7)	
MS4	171 (95.0)	Other	0 (0.0)	
Other	9 (5.0)			

### Survey Results

#### Applicant Survey

**Figure 1.**
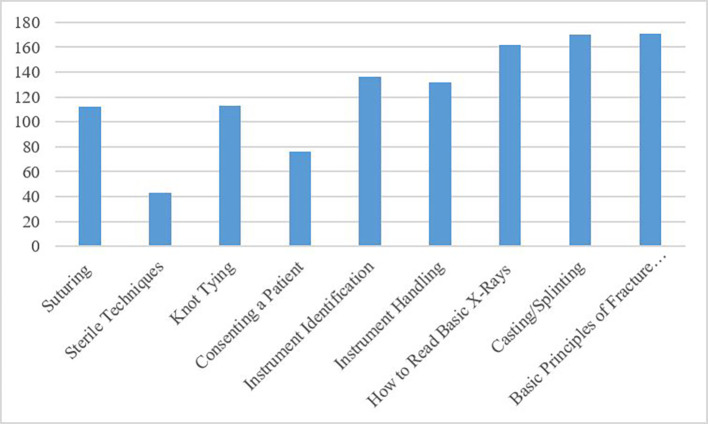
Skills Identified as Being Important by Applicants

170 out of 181 (94%) of respondents indicated that their medical school did not offer an orthopaedic surgical skills course during the 4 ^th^ year of medical school. Eleven applicants (6%) had a skills course offered at their medical school and all of these students had or planned to take part in the course. One of the eleven (9%) applicants indicated that he or she had yet to participate in the course. Among the 10 respondents who had participated in the orthopaedic surgical skills curriculum at their institution, 9 (90%) believed that the course better prepared them for an orthopaedic surgery residency. 160 of 171 (94%) of applicant respondents agreed that implementing an orthopaedic surgical skills course into the 4 ^th^ year medical school curriculum would be beneficial. Applicants rated basic principles of fracture reduction (95%; 171/180), casting/splinting (94%; 170/180), reading basic x-rays (90%; 162/180), and instrument identification (76%; 136/180) as the most important components to be included in such a curriculum ( Figure 1).

Applicants Indicating Importance of Specific Skills: Applicants indicated which skills should be included in an orthopaedic surgical skills curriculum to improve their performance prior to entering residency

Additionally, 48 out of 180 applicants (27%) indicated that an orthopaedic surgical skills course should be structured as four to six 1-hour sessions spread out over 4 weeks. Other structures for such a course that applicants felt would be appropriate were four to six 1-hour sessions spread out over 1 week (22%; 40/180), and one full day from 8a-4p (18%; 32/180) ( Table 2). Of note, 13 applicants made suggestions for how to structure a surgical skills course with pertinent examples seen in Table 3.

**Table 2.** Applicants’ opinion on how an orthopaedic surgical skills course should be structured.

Course Structure	Number of Applicants (%)
One Full Day (8a-4p)	32 (17.8)
One Half Day (8a-12p)	8 (4.4)
Four to six 30-minute sessions spread out over 1 week	6 (3.3)
Four to six 1-hour sessions spread out over 1 week	40 (22.2)
Four to six 30 minute sessions spread out over 4 weeks	12 (6.7)
Four to six 1-hour sessions spread out over 4 weeks	48 (26.7)
Four to six 30 minute sessions spread out over the academic year	1 (0.6)
Four to six 1-hour sessions spread out over the academic year	20 (11.1)
Other	13 (7.2)

**Table 3.** Applicant and Resident “Other” Suggestions for the Structure of an Orthopaedic Surgical Skills Course.

Applicants	Residents
Four 1 hour sessions per month for 6 months	One week
Anything would be beneficial, but it should only be offered after the match for 4 ^th^ year students. This way no time is wasted and no spots in the curriculum/course are wasted.	None. This is learned on the job. Students are more benefited from learning sterile technique. The point of surgical residency is to learn the skills. This is outside the realm of medical school, where you are learning other important things and not supposed to be focused on specialty needs.
2-3 one hour blocks per week for one month.	
Half days for two weeks.	
2-3 full days; gaining a regular schedule (e.g. 4 meetings over a month) is challenging with 4 ^th^ year schedules.	
A one month course.	

#### Resident Survey

**Figure 2.**
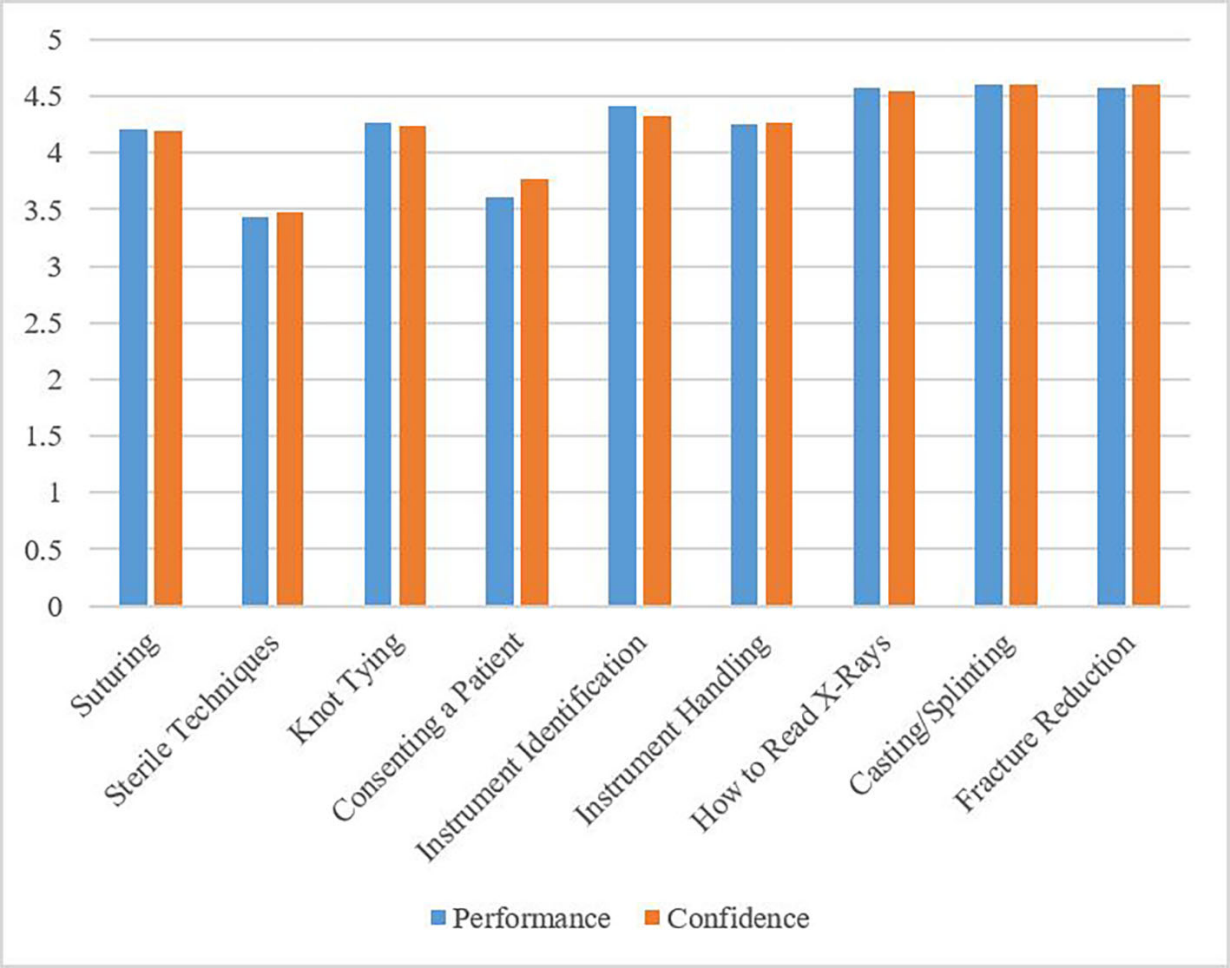
Orthopaedic Resident Confidence and Performance

41 of 117 (35%) residents indicated that they participated in a surgical skills course prior to residency, although an orthopaedic specific course was not listed as a response. Residents strongly agreed that taking a basic skills course in reading x-rays (77/117; 66%), casting/splinting (83/117; 71%), and fracture reduction (82/117; 70%) would have improved their performance during the first two years of residency. Additionally, residents strongly agreed that a course in reading x-rays (72/117; 62%), casting/splinting (78/117; 67%), and fracture reduction (78/117; 67%) would have improved their confidence during the junior years of residency. Other skills that residents suggested would have been improved had they participated in a skills course included instrument identification, instrument handling, suturing, and knot tying ( Figure 2).

Resident Confidence and Performance: Residents indicated whether or not a course in each specific skill would have improved their confidence and/or performance during residency. On a Likert scale of 1-5, 1 indicates “strongly disagree,” 2 is “disagree,” 3 represents “neutral,” 4 is “agree,” and 5 represents “strongly agree.”

110 of 118 junior residents (93%) believed implementing an orthopaedic surgical skills course during the 4 ^th^ year of medical school would have been beneficial. Most residents indicated that such a course should either be structured as four to six 1-hour sessions spread throughout 4 weeks (34%; 39/115) or as four to six 1-hour sessions spread out over 1 week (31%; 30/115). Additionally, 2 residents provided “other” suggestions for an effective course structure ( Table 1).

## Discussion

Our study demonstrated that applicants and junior orthopaedic surgery residents believe that an orthopaedics specific surgical skills course would improve both confidence and performance during the first two years of residency. Directly surveying current and future orthopaedic surgeons provided this study with a baseline consensus, laying the groundwork for the development of future surgical skills pilot programs. These results were similar to those of previous studies in general surgery. The general surgery surgical skills pilot programs implemented in the 4 ^th^ year curriculum at 50 US allopathic medical schools have improved junior resident confidence and performance in surgical skills ( Naylor *et al.,* 2010), ( Peyre *et al.,* 2006). Such a program has not been established for students beginning careers in orthopaedic surgery. However, previously implemented surgical skills courses for junior orthopaedic surgery residents have been viewed favorably ( Butler *et al.,* 2017), ( Ford, Patt and Scannell, 2016), ( Sonnadara *et al.,* 2011).

In 2013, Karam *et al*. distributed online surveys to both orthopaedic surgery residency program directors and residents to assess the interest in an orthopedic surgery skills course ( Karam *et al.,* 2013). The surveys were sent to 185 program directors (PDs) and 4,549 residents with 86 of 185 (46%) and 687 of 4,549 (15%) responding, respectively. Of participants, 80% of program directors and 86% of residents agreed that surgical skills simulations should become a required part of training. Furthermore, 82% of program directors and 76% of residents were interested in a standardized surgical skills curriculum. The most cited barrier to the implantation of such a course was lack of funding.6 The results of our study are similar, in that 110 of 118 (93%) residents believe in the implementation of a surgical skills program during the 4th year of medical school.

Previous studies have demonstrated that there is a strong interest in the development and analysis of orthopaedic specific surgical skills programs. At the University of Iowa, authors developed and implemented a month-long, standard curriculum for six PGY-1 residents, which consisted of 31 modules, each taking residents approximately six hours to complete ( Karam *et al.,* 2013). At the end of the month, residents were given questionnaires to rate their experience. Of participants, 83% reported that the program improved their surgical skills and would enhance their operating room safety. In addition, 100% of the participants agreed that the course improved their orthopaedic training and felt that such a program should be a permanent fixture in resident education. The total cost for the month was approximately $22,500 ( Karam *et al.,* 2013).

Similarly, Westerlind *et al*. developed a one month surgical skills training program for PGY-1 general surgery residents ( Westerlind *et al.,* 2014). During the entire month, six PGY-1 residents worked full time on the surgical skills course and were free of clinical responsibilities. Eighteen separate skills topics formed the framework of the month-long curriculum. The six PGY-1 residents who completed the course indicated that their surgical skills improved after the course and 86% believed the course would enhance their operating room safety. 100% of residents felt the course improved their orthopaedic training and that such a course should be a permanent part of surgical education. The total cost of their month-long program was $21,864 ( Westerlind *et al.,* 2014). Ford *et al*. and Sonnadara *et al*. describe the incorporation of similar programs at their respective institutions, with variable results ( Ford, Patt and Scannel, 2016), ( Sonnadara *et al.,* 2011). Course details for each orthopaedic surgical skills pilot program offered to current residents are outlined in Table 4. Our study demonstrated that orthopaedic applicants, 4 ^th^ year medical students, should be a target group as well.

**Table 4.** Summary of Orthopaedic Surgery Surgical Skills Courses.

Authors	Participants	Description	Results	Cost Per Course
Karam *et al.*	PGY-1 Residents	19 individual modules over 1 month	100% resident agreement that curriculum enhanced orthopaedic training	$22,500
Westerlind *et al.*	PGY-1 Residents	19 individual modules over 1 month	93% comprehensive resident satisfaction	$21,864
Ford *et al.*	PGY-1 Residents	Multiple modules totaling 89 hours	Every session was rated as at least “good” (98%), with 32% of modules achieving an “excellent” rating.	$8,100
Sonnadara *et al.*	PGY-1 Residents	Randomized controlled trial with study group undergoing a 30-day intensive surgical skills course. Control groups continued with standard orthopaedic residency curriculum (on and off-service)	Study group performed significantly better on post course assessment than control groups (ISL-on-service difference = 7.648, P < .05; ISL-off-service difference 9.161, P < .01)	N/A

Applicant and resident respondents had similar ideas regarding which skills should be included in an orthopaedic-specific surgical skills course. Both groups suggested that the most important skills to be included in the curriculum should be basic principles of fracture reduction, casting/splinting, reading basic x-rays, and instrument identification. Suturing, knot tying, consenting a patient, and sterile technique were deemed less important by both applicant and resident respondents. The skills rated lower by the respondents are important for future orthopaedic surgery residents to master, but are likely learned during the 3 ^rd^ year clerkships and perfected during residency. Excluding these from a 4 ^th^ year skills course may decrease the costs of these programs to medical schools. Additionally, a 2018 study performed in the UK by Bennett et.al., demonstrated that medical student-to-medical student peer-assisted learning was an effective and feasible method for teaching surgical skills and improved confidence among healthcare undergraduates; possibly a less expensive bridge to an expansive orthopaedic surgical skills program ( Bennett, Morris and Mirza, 2018).

There are several limitations to this study. First, given the low response rate from the junior orthopaedic surgery residents it is not possible to make definitive conclusions about their opinions on incorporating a surgical skills curriculum into the 4 ^th^ year of medical school. Busy resident schedules, a heavy survey burden, and changes in resident email addresses are all possible contributing factors to this low response rate. While the response rate was low, the data obtained was in line with the data received from the applicants. Second, this was a survey-based study and is subject to all of the inherent limitations of this study design. We acknowledge that there exists a certain level of variability in how questions and responses are interpreted by both respondents and survey administrators. Additionally, a response bias may exist, as residents and applicants could choose to respond to various questions. Because the survey did not require a response to move on to the next question, there was an uneven number of responses to the different questions. Future research should evaluate the cost of an orthopaedic surgical skills curriculum to medical schools. It will be important to understand whether medical schools (or orthopaedic departments specifically) are willing to pay for such a program, as this is a major determining factor in the viability of a surgical skills course for 4 ^th^ year medical students entering orthopaedic surgery residency.

## Conclusion

Currently, there is no standardized surgical skills curriculum for medical students pursuing a career in orthopaedic surgery. Pilot programs should be developed to optimize and, eventually, standardize an orthopaedic preparatory course. Both current residents and applicants believed that such a program would benefit junior residents by increasing confidence, performance, and preparedness for an orthopaedic surgery residency.

## Take Home Messages

Current residents and medical students agree that implementing an orthopaedic specific surgical skills course into the curriculum of the 4th year of medical school would be beneficial to confidence and performance as a junior resident.

## Notes On Contributors

• Lucy E. Meyer is a 4th year medical student at Tulane University School of Medicine.
• Ocean V. Thakar is a 1st year orthopaedic surgery resident at MedStar Union Memorial Hospital in Maryland. He attended Drexel University College of Medicine at the time this project was performed.
• Symone M. Brown is a research assistant to Dr. Mary Mulcahey at Tulane University School of Medicine.
• Jeffrey D. Trojan is a 2nd year medical student at Tulane University School of Medicine.
• Dr. Mary K. Mulcahey is an assistant professor of orthopaedic surgery at Tulane University School of Medicine.

## Declarations

The author has declared that there are no conflicts of interest.

## Ethics Statement

IRB was obtained from Tulane University School of Medicine under the approval/reference number 2017-815. Ethical guidelines conducted utilizing the Survey Ethics section of the Encyclopedia of Survey Research Methods. These guidelines state, “these procedures are essential to the research process so that explicit care is taken that (a) no harm is done to any survey respondent, and (b) no survey respondent is unduly pressured or made to feel obligated to participate in a survey.” Lavrakas, P. J. (2008). Encyclopedia of survey research methods Thousand Oaks, CA: Sage Publications, Inc. doi: 10.4135/9781412963947.

## External Funding

This article has not had any External Funding

## References

[ref1] American Board of Surgery (2014) Statement on surgical preresidency preparatory courses. The American surgeon. 80(11), pp.1085–1086. Available at: http://www.ncbi.nlm.nih.gov/pubmed/25347496( Accessed: 14 November 2019). 25347496

[ref2] Bennett S. R. Morris S. R. and Mirza S. (2018) Medical Students Teaching Medical Students Surgical Skills: The Benefits of Peer-Assisted Learning. Journal of Surgical Education. Elsevier Inc.,75(6), pp.1471–1474. 10.1016/j.jsurg.2018.03.011 29653841

[ref3] Butler B. A. (2017) Simulation-Based educational module improves intern and medical student performance of closed reduction and percutaneous pinning of pediatric supracondylar humeral fractures. Journal of Bone and Joint Surgery - American Volume. Lippincott Williams and Wilkins. 99(23), p. e128. 10.2106/JBJS.17.00425 29206799

[ref4] Debas H. T. (2005) American Surgical Association Blue Ribbon Committee report on surgical education: 2004. Annals of Surgery.pp.1–8. 10.1097/01.sla.0000150066.83563.52 PMC1356839 15621984

[ref5] Dougherty P. J. and Marcus R. E. (2013) ACGME and ABOS changes for the orthopaedic surgery PGY-1 (intern) year. Clinical Orthopaedics and Related Research. Springer New York LLC. 471(11), pp.3412–3416. 10.1007/s11999-013-3227-9 23934034 PMC3792253

[ref6] Ford S. E. Patt J. C. and Scannell B. P. (2016) A comprehensive, high-quality orthopedic intern surgical skills program. Journal of Surgical Education. Elsevier Inc.,73(4), pp.553–558. 10.1016/j.jsurg.2016.03.008 27142722

[ref7] Karam M. D. (2013) Current and future use of surgical skills training laboratories in orthopaedic resident education: A national survey. Journal of Bone and Joint Surgery - Series A. 10.2106/JBJS.L.00177 23283381

[ref8] Karam M. D. (2013) Development of an orthopaedic surgical skills curriculum for post-graduate year one resident learners - the University of Iowa experience. The Iowa orthopaedic journal. 33, pp.178–184. 24027480 PMC3748876

[ref9] Miller R. (2016) Introducing a fresh cadaver model for ultrasound-guided central venous access training in undergraduate medical education. Western Journal of Emergency Medicine. eScholarship. 17(3), pp.362–366. 10.5811/westjem.2016.3.30069 27330672 PMC4899071

[ref10] Naylor R. A. (2010) Preparing medical students to enter surgery residencies. American Journal of Surgery. 199(1), pp.105–109. 10.1016/j.amjsurg.2009.09.003 20103074

[ref11] Peyre S. E. (2006) A Surgical Skills Elective Can Improve Student Confidence Prior to Internship. Journal of Surgical Research. 133(1), pp.11–15. 10.1016/j.jss.2006.02.022 16580692

[ref12] Sonnadara R. R. (2011) Orthopedic boot camp: Examining the effectiveness of an intensive surgical skills course. Surgery. 149(6), pp.745–749. 10.1016/j.surg.2010.11.011 21236456

[ref13] Successfully Navigating the First Year of Surgical Residency (2005). Available at: https://www.facs.org/education/division-of-education/publications/navigatefirstyear( Accessed: 22 April 2017).

[ref14] Teo A. R. (2011) The key role of a transition course in preparing medical students for internship. Academic Medicine. Lippincott Williams and Wilkins. 86(7), pp.860–865. 10.1097/ACM.0b013e31821d6ae2 PMC3128667 21617513

[ref15] Tocco N. (2013) Innovation in internship preparation: An operative anatomy course increases senior medical students’ knowledge and confidence. American Journal of Surgery. 206(2), pp.269–279. 10.1016/j.amjsurg.2012.07.043 23433887

[ref16] Westerlind B. (2014) A surgical skills training curriculum for PGY-1 residents AAOS exhibit selection. Journal of Bone and Joint Surgery - American Volume. Journal of Bone and Joint Surgery Inc. 96(16), p.e140.1–e140.6. 10.2106/JBJS.M.01414 PMC4574912 25143508

[ref17] Zeng W. Woodhouse J. and Brunt L. M. (2010) Do preclinical background and clerkship experiences impact skills performance in an accelerated internship preparation course for senior medical students? Surgery. 148(4), pp.768–777. 10.1016/j.surg.2010.07.022 20705307

