# Supplementary material for: Surgical Skills Course for Fourth Year Medical Students Entering an Orthopaedic Surgery Residency
Source: MedEdPublish (2016). 2020 Feb 19;9:34. [Version 1] doi: 10.15694/mep.2020.000034.1 (PMC10697433; doi:10.15694/mep.2020.000034.1)
Supplement: Peer review history

# Reviewer report: Reviewer response for version 1

**DOI:** 10.21956/mep.19960.r30326

**Published:** 2020-05-19

**Authors:** Trevor Gibbs

**Affiliations:** 1 AMEE

This review has been migrated. The reviewer awarded 4 stars out of 5 This was a very well researched paper, very clear and well written and certainly shows that the authors had researched the subject extensively. I thought that they explained the positive and negative aspects of their work well.However, to me it felt like the work up for a more important piece of work which related to the development of a much needed course and its evaluation- a more valuable piece of educational work that would have greater impact.Despite my reservations, I would recommend that this paper is read by all those involved with surgical curricula development.

Reviewer Expertise:

NA

No decision status is available

# Reviewer report: Reviewer response for version 1

**DOI:** 10.21956/mep.19960.r30327

**Published:** 2020-02-19

**Authors:** Julie Hunt

**Affiliations:** 1 Lincoln Memorial University

This review has been migrated. The reviewer awarded 3 stars out of 5 The authors report the results of a survey of fourth year medical students and PGY 1 and 2 orthopedic surgical residents which asks about the potential addition of a surgical skills course during the fourth year of medical school. Despite a somewhat predictably low response rate, the overall number of responses was adequate for drawing conclusions. The study probably gives a reasonably accurate estimation of how many students have already taken an orthopedic surgical skills course during medical school (6%), but it is likely less to be accurate at predicting how many medical students would like to take an orthopedic surgical course, as the surveyed students are those who have already applied to an orthopedic surgical residency program. Surveying those interested in a particular specialty will nearly always result in them requesting more training in their area of interest; the problem is finding curricular time to accommodate everyone's areas of interest.I think this article is a helpful read for those in this specialty field, as it suggests the specific tasks that medical students would like to have included in an orthopedic surgical skills course. I agree with the authors that such a course would better prepare students for starting an orthopedic surgery residency, if time could be found for it in the medical school curriculum.

Reviewer Expertise:

NA

No decision status is available
